# Cholesterol efflux capacity is increased in subjects with familial hypercholesterolemia in a retrospective case–control study

**DOI:** 10.1038/s41598-023-35357-4

**Published:** 2023-05-24

**Authors:** Juana Maria Sanz, Andrea D’Amuri, Domenico Sergi, Sharon Angelini, Valeria Fortunato, Elda Favari, Giovanni Vigna, Giovanni Zuliani, Edoardo Dalla Nora, Angelina Passaro

**Affiliations:** 1 Department of Chemical, Pharmaceutical and Agricultural Sciences, University of Ferrara, Via Luigi Borsari, 46, 44121 Ferrara, Italy; 2 Medical Department, University Hospital of Ferrara Arcispedale Sant’Anna, Via Aldo Moro, 8, 44124 Cona, Ferrara Italy; 3 Department of Translational Medicine, University of Ferrara, Via Luigi Borsari, 46, 44121 Ferrara, Italy; 4 Department of Food and Drug, University of Parma, Viale delle Scienze 27/A, 43124 Parma, Italy; 5 Medicina Generale, Ospedale di Trecenta, Via U. Grisetti, 265, 45027 Trecenta, RO Italy; 6 Research and Innovation Section, University Hospital of Ferrara Arcispedale Sant’Anna, Via Aldo Moro, 8, 44124 Cona, Ferrara Italy

**Keywords:** Biochemistry, Diseases, Cardiovascular diseases, Metabolic disorders

## Abstract

Familial Hypercholesterolemia (FH) is characterized by an increase in Low-Density Lipoprotein Cholesterol (LDL-C) and by premature Cardiovascular Disease (CVD). However, it remains to be fully elucidated if FH impairs cholesterol efflux capacity (CEC), and whether CEC is related to lipoprotein subfraction distribution. This study aimed at comparing FH patients and age, sex and BMI matched controls in terms of LDL and HDL subfraction distribution as well as CEC. Forty FH patients and 80 controls, matched for age, sex and BMI, were enrolled in this case–control study. LDL and HDL subfractions were analyzed using the Quantimetrix Lipoprint System. CEC was evaluated as aq-CEC and ABCA1-CEC. FH subjects showed a significantly higher concentration of all LDL subfractions, and a shift from large to small HDL subfraction pattern relative to controls. FH subjects with previous CVD event had smaller LDL lipoproteins than controls and FH subjects without previous CVD event. Both aq-CEC and ABCA1-CEC were increased in FH patients with respect to controls. To conclude, FH subjects had a metabolic profile characterized not only by higher LDL-C but also by shift from large to small HDL subfraction phenotype. However, FH subjects showed an increase CEC than controls.

## Introduction

Familial hypercholesterolemia (FH) is a common inherited dominant autosomal disorder caused by genetic mutations in genes that encode for Low Density Lipoprotein (LDL) particles clearance^1^. FH is characterized by high levels of Low Density Lipoprotein Cholesterol (LDL-C) and susceptibility to an early onset of Cardiovascular Disease (CVD)^1,2^ Numerous reports have consistently demonstrated a long-linear relationship between the change in plasma LDL-C concentration and the risk of CVD^3^. However, besides total LDL-C amount, it seems that also lipoprotein characteristics could influence atherogenic properties of LDL-C. In particular, concentration of small-dense LDL (sdLDL) was more closely associated with the incidence of CVD than large buoyant LDL (lbLDL)^4,5^.

High Density Lipoproteins (HDL), generally considered as atheroprotective particles, are involved in cholesterol transport from peripheral tissues to the liver, a mechanism known as Reverse Cholesterol Transport (RCT), and also exert antioxidant and anti-inflammatory activities^6,7^. HDL-mediated efflux of cholesterol from peripheral cells (Cholesterol Efflux Capacity, CEC) is a critical metric of HDL functionality and cardiovascular protection from atherosclerosis^8^ as well as a valuable target for CVD prevention^9–11^. Interestingly, HDL particle size may affect CEC, suggesting that differences in HDL efflux capacity may be due to structural differences in HDL particles^12,13^. A lot of evidence suggests that altered RCT could further increase CVD progression in FH subjects^14^. Some authors have observed a CEC impairment in FH subjects^15^ correlating with CVD in these patients^16^.

Although it is well known that FH subjects have a show pronounced CVD risk, it is yet poorly understood if they present a particular lipoprotein subclasses distribution or if lipoprotein subclasses sizes could influence CEC and incidence of CVD events in FH subjects.

Hence, we performed a case–control study to compare LDL and HDL subclasses distribution and their association with CEC in a cohort of FH subjects with or without cardiovascular events compared to controls.

## Results

### Baseline characteristics

Baseline clinical characteristics of subjects are summarized in Table 1. No significant differences with regard to the prevalence of hypertension and obesity were detected between control and FH group. There was an obvious significant difference between FH and the control group in terms of CVD history and use of lipid lowering drugs, with both variables being higher in FH group (Table 1).

**Table 1:** Clinical characteristics of subjects by group.

	Controls		FH		P value
Subjects	80		40		
Female N (%)	48 (60.0%)		24 (60.0%)		0.580
Hypertension N (%)	32 (40.0%)		16 (40.0%)		1.000
Obesity N (%)	8 (10.0%)		6 (15.0%)		0.291
CVD N (%)	0 (0.0%)		11 (27.5%)		**< 0.001**
Lipid-lowering drug-treated N (%)	0 (0.0%)		33 (82.5%)		** < 0.001**
Smoker N (%)	39 (49.5%)		11 (22.2%)		**0.022**

	Mean ± SD	Median (Q1–Q3)	Mean ± SD	Median (Q1–Q3)	P value
Age (years)	53 ± 12	54 (43–63)	53 ± 12	54 (42–63)	0.701^§^
BMI (Kg/m^2^)	25.3 ± 3.9	24.6 (22.3–27.9)	25.9 ± 3.9	26.1 (22.9–27.8)	0.312^§^
SBP (mmHg)	131 ± 15	130 (122–140)	129 ± 16	128 (120–134)	0.137^§^
DBP (mmHg)	82 ± 12	80 (74–90)	82 ± 9	80 (77–90)	0.345^§^

Bold values indicate statistically significant results.

*FH* Familial Hypercholesterolemia, *CVD* CardioVascular Disease, *BMI* Body Mass Index, *SBP* Systolic Blood Pression, *DBP* Diastolic Blood Pression, *SD* Standard Deviation. Categorical variables were analyzed with Fisher Test. Means compared by Student's t-test, whereas medians from not normally distributed variables, were compared using the nonparametric Mann Whitney test^§^.

### Lipid profile and lipoprotein subfraction

**Figure 1:**
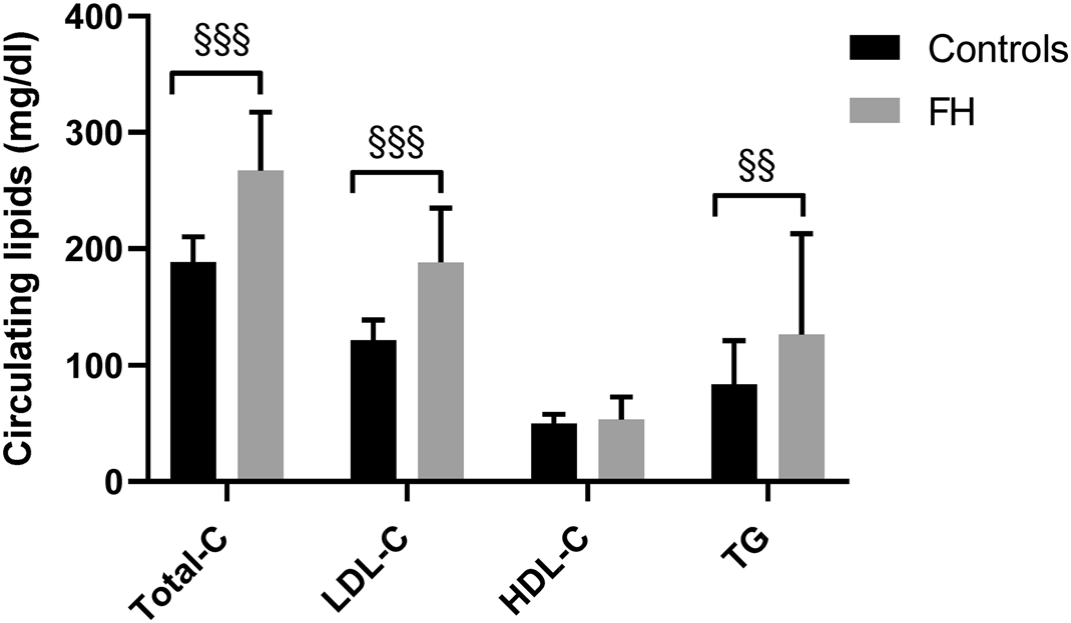
Circulating lipid profile in controls and FH subjects. Data are reported as mean ± standard deviation. *FH* Familial Hypercholesterolemia, *Total-C* total cholesterol, *LDL-C* Low Density Lipoprotein Cholesterol, *HDL-C* High Density Lipoprotein Cholesterol, *TG* Triglycerides. Means were compared by Student's t-test, whereas the medians of not normally distributed variables (Total-C, LDL-C, TG), were compared using the nonparametric Mann Whitney test^§^. ^§§^p < 0.01; ^§§§^p < 0.001.

As expected, serum concentrations of TC, LDL-C and triglycerides were higher in FH subjects compared to the controls. There were no differences in HDL-C between groups (Fig. 1).

FH subjects showed higher levels of sdLDL and lbLDL relative to controls. However, sdLDL to total LDL ratio was not different between the two groups, whereas lbLDL percentage showed a trend towards higher values FH group compared to controls (p = 0.055) (Table 2).

**Table 2:** LDL and HDL subfraction in controls and FH subjects.

	Controls		FH		P value
	Mean ± SD	Median (Q1–Q3)	Mean ± SD	Median (Q1–Q3)	
sdLDL (%)	5.5 ± 5.5	3.8 (1.9–7.0)	7.5 ± 8.8	4.2 (2.8–7.7)	0.370^§^
lbLDL (%)	58.6 ± 6.9	59.4 (53.3–63.3)	55.5 ± 10.4	57.7 (52.1–62.0)	0.055
sdLDL (mg/dl)	6.2 ± 6.9	4.5 (2.3–7.9)	13.8 ± 19.8	7.5 (3.9–15.6)	**0.002**^§^
lbLDL (mg/dl)	64.6 ± 12.7	64.0 (57.6–74.1)	98.8 ± 28.1	95.9 (78.4–120.0)	** < 0.001**
LDL size (Å)	268.4 ± 4.1	269.0 (266.0–271.3)	267.1 ± 5.3	268.0 (266.0–271.0)	0.330^§^
l-HDL (%)	33.9 ± 6.2	34.6 (28.9–38.4)	30.5 ± 11.2	31.2 (23.8–38.6)	**0.037**
m-HDL (%)	45.4 ± 3.8	45.5 (43.5–48.1)	46.2 ± 4.4	46.3 (42.6–49.1)	0.355
s-HDL (%)	20.6 ± 4.7	19.6 (17.0–24.2)	23.3 ± 9.1	23.3 (17.3–30.9)	**0.039**
l-HDL (mg/dl)	17.1 5.0	17.1 (13.4–20.0)	17.2 ± 10.6	14.7 (9.0–22.9)	0.974
m-HDL (mg/dl)	22.6 ± 3.4	21.9 (20.1–24.9)	24.5 ± 8.1	24.0 (18.1–28.1)	0.077
s-HDL (mg/dl)	10.2 ± 2.4	10.0 (8.4–11.6)	11.9 ± 4.7	12.1 (9.1–14.7)	**0.010**

Bold values indicate statistically significant results.

*FH* Familial Hypercholesterolemia, *LDL* Low Density Lipoprotein, *HDL* High Density Lipoprotein, *sdLDL* small dense LDL, *lbLDL* large buoyant LDL, *l-HDL* large HDL, *m-HDL* medium HDL, *s-HDL* small HDL, *SD* Standard Deviation. Means compared by Student's t-test, whereas medians from not normally distributed variables, were compared using the nonparametric Mann Whitney test^§^.

Concerning HDL subfractions, FH subjects showed significantly higher levels of s-HDL either if expressed as absolute values or in percentage, (Table 2). On the contrary, percentage, but not absolute values of l-HDL, was lower in FH subjects than in control group (Table 2).

**Figure 2:**
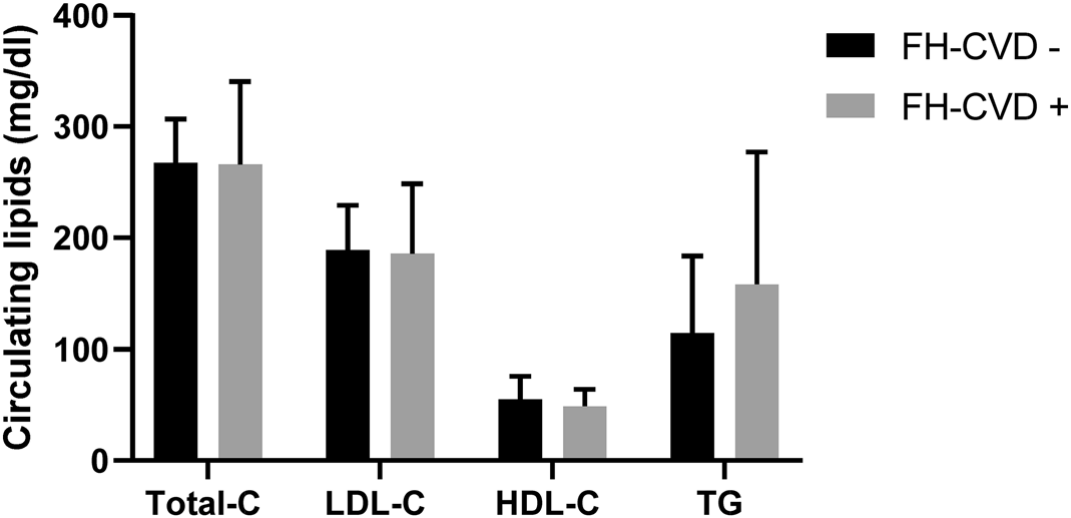
Lipid profile in FH subjects without or with a previous CVD event. Data are reported as mean ± standard deviation. *FH* Familial Hypercholesterolemia, *Total-C* total cholesterol, *LDL-C* Low Density Lipoprotein Cholesterol, *HDL-C* High Density Lipoprotein Cholesterol, *TG* Triglycerides, *CVD* CardioVascular Disease. The medians of not normally distributed variables (Total-C, LDL-C, HDL-C TG), were compared using the nonparametric Mann Whitney test§. FH-CVD−, N = 29; FH-CVD+, N = 11.

When FH subjects were stratified according to the presence (FH-CVD+) or absence (FH-CVD−) of CVD history, there was no difference in lipid profile or LDL and HDL subfraction between FH-CVD− and FH-CVD+, except for mean LDL size that was smaller in FH-CVD+ than FH-CVD− (Fig. 2 and Table 3).

**Table 3:** Lipid profile in FH subjects without or with a previous CVD event.

	FH-CVD−		FH-CVD+		P value
	Mean ± SD	Median (Q1–Q3)	Mean ± SD	Median (Q1–Q3)	
sdLDL (%)	6.2 ± 6.4	3.7 (2.8–7.6)	10.7 ± 13.1	4.9 (2.7–16.5)	0.637§
lbLDL (%)	56.1 ± 9.6	57.8 (51.6–60.9)	54.1 ± 12.5	54.4 (52.2–62.9)	1.000
sdLDL (mg/dl)	10,7 ± 9,6	7.1 (4.4–15.5)	22.0 ± 34.2	8.5 (3.8–25.6)	0.644§
lbLDL (mg/dl)	101,1 ± 29,1	103.9 (79.0–120.3)	92.6 ± 25.2	87.1 (69.8–110.1)	0.640
LDL size (Å)	267.8 ± 4.3	269.0 (266.0–271.0)	265.4 ± 7.4	267.0 (261.0–271.0)	**0.046§**
l-HDL (%)	31.3 ± 10.2	33.2 (24.4–39.1)	28.4 ± 13.9	26.6 (14.9–38.9)	0.891
m-HDL (%)	46.1 ± 4.3	46.4 (42.5–48.8)	46.3 ± 4.6	45.0 (42.5–50.1)	0.999
s-HDL (%)	22.5 ± 8.7	22.9 (16.4–28.7)	25.3 ± 10.0	23.4 (18.9–33.9)	0.807
l-HDL (mg/dl)	17.9 ± 10.4	16.3 (10.8–19.8)	15.3 ± 11.2	17.9 ± 10.4	0.883
m-HDL (mg/dl)	25.3 ± 8.8	24.1 (18.2–29.7)	22.1 ± 5.8	25.3 ± 8.8	0.466
s-HDL (mg/dl)	12.1 ± 5.1	11.9 (9.2–16.1)	11.2 ± 3.5	12.1 ± 5.1	0.884

Bold values indicate statistically significant results.

*FH* Familial Hypercholesterolemia, *LDL* Low Density Lipoprotein, *HDL* High Density Lipoprotein, *sdLDL* small dense LDL, *lbLDL* large buoyant LDL, *l-HDL* large HDL, *m-HDL* medium HDL, *s-HDL* small HDL, *CVD* CardioVascular Disease, *SD* Standard Deviation. Means compared by Student's t-test, whereas medians from not normally distributed variables, were compared using the nonparametric Mann Whitney test^§^. FH-CVD−, N = 29; FH-CVD+, N = 11.

Considering previous studies^17^ suggested hypocholesterolemic therapy affecting circulating LDL size, in our cohort we also performed a subgroup analysis to compare naïve versus patients on a lipid lowering therapy. As expected, lipid-lowering drug-treated patients had lower levels of TC and LDL-C than naïve patients (Supplementary Table 1S). This effect was due to a lower absolute concentration of lbLDL, while these groups did not differ in sdLDL concentration or LDL size (Supplementary Table 2S).

Both groups, naïve and lipid-lowering drugs treated patients had similar levels of triglycerides, HDL-C concentrations or HDL-C subfraction, except for the percentage of m-HDL subfraction that was higher in the treated versus naïve FH patients (Supplementary Tables 1S and 2S).

### Cholesterol efflux capacity in controls and FH subjects with or without CVD

**Figure 3:**
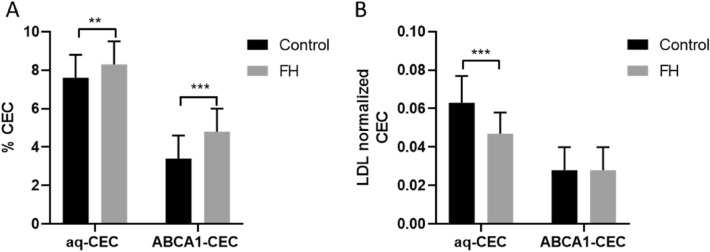
Cholesterol Efflux Capacity in controls and FH subjects. Data are reported as mean ± standard deviation. (**A**) % CEC, (**B**) LDL-normalized CEC. *FH* Familial Hypercholesterolemia, *aq-CEC* aqueous diffusion Cholesterol Efflux Capacity, *ABCA1-CEC* ATP Binding Cassette Cholesterol Efflux Capacity, *LDL-C normalized aq-CEC* Low Density Lipoprotein normalized aqueous diffusion Cholesterol Efflux Capacity, *LDL-C normalized ABCA1-CEC* Low Density Lipoprotein normalized ATP Binding Cassette Cholesterol Efflux Capacity. Means were compared by Student's t-test. **p < 0.01; ***p < 0.001.

**Figure 4:**
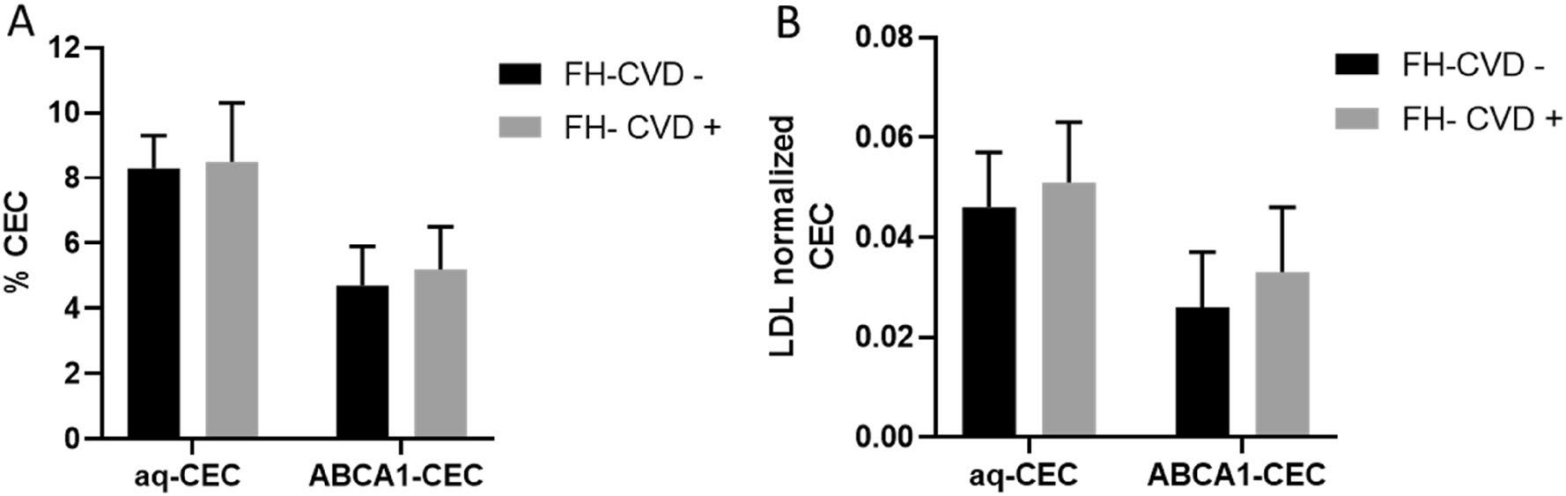
Cholesterol Efflux Capacity in FH subjects without or with a previous CVD event. Data are reported as mean ± standard deviation *FH* Familial Hypercholesterolemia, *aq-CEC* Aqueous diffusion Cholesterol Efflux Capacity, *ABCA1* ATP Binding Cassette A1, *CEC* Cholesterol Efflux Capacity, *CVD* CardioVascular Disease, *SD* Standard Deviation. Means compared by Student's t-test. FH-CVD−, N = 29; FH-CVD+, N = 11.

The HDL of FH subjects showed an increased CEC than controls, both via aq-CEC and ABCA1-CEC (Fig. 3). However, when CEC values were normalized for LDL-C, aq-CEC was significantly higher in the control than the FH group, while there were no differences in LDL-C normalized ABCA1-mediated CEC between the groups (Fig. 3). CVD status nor the normalization for LDL affected CEC or LDL-C normalized CEC (Fig. 4).

Analysis of CEC did not showed differences between naïve or lipid-lowering drug-treated patients, except for LDL-C normalized aq-CEC that was lower in naïve patients (Supplementary Table 3S).

Analyzing the overall population, aq-CEC positively correlated with TC, triglycerides, LDL-C, sdLDL-C and lbLDL-C and was inversely associated with LDL particle size. There was no relationship between HDL-C or HDL subclasses and aq-CEC. ABCA1-CEC, on the other hand, was positively correlated with TC, LDL-C and lbLDL-C, while there were no association between ABCA1-CEC and triglycerides, sdLDL-C, LDL size, HDL-C or HDL subclasses (Table 4).

**Table 4:** Correlation analysis between Cholesterol Efflux Capacity and lipidomic parameters from all subjects.

	aq-CEC (%)		ABCA1-CEC (%)	
	Pearson’s r	P value	Pearson’s r	P value
Total-C (mg/dl)	0.376	** < 0.001**	0.317	**0.001**
LDL-C (mg/dl)	0.346	** < 0.001**	0.298	**0.002**
HDL-C (mg/dl)	− 0.005	0.959	0.102	0.300
TG (mg/dl)	0.335	** < 0.001**	0.133	0.175
sdLDL (mg/dl)	0.229	**0.020**	0.120	0.231
lbLDL (mg/dl)	0.206	**0.037**	0.247	**0.012**
LDL size (Å)	− 0.243	**0.013**	− 0.081	0.411
l-HDL (mg/dl)	− 0.025	0.801	0.038	0.697
m-HDL (mg/dl)	0.035	0.721	0.103	0.297
s-HDL (mg/dl)	− 0.021	0.829	0.138	0.160

Bold values indicate statistically significant results.

*aq-CEC* Aqueous diffusion Cholesterol Efflux Capacity, *Total-C* total cholesterol, *LDL-C* Low Density Lipoprotein Cholesterol, *HDL-C* High Density Lipoprotein Cholesterol, *TG* Triglycerides, *sdLDL* small dense LDL, *lbLDL* large buoyant LDL, *l-HDL* large HDL, *m-HDL* medium HDL, *s-HDL* small HDL, *Pearson’s r* Pearson correlation coefficient.

When focusing in particular on FH subjects, aq-CEC was only directly associated with TC and triglycerides; there were no associations between aq-CEC or ABCA1-CEC and lipid profile or lipoprotein subfractions (Supplementary Table 4S).

## Discussion

The results of this case–control study provided further evidence in support the impaired cardiometabolic profile of FH subjects. Indeed, these individuals showed higher levels of TC, LDL-C, sdLDL-C, lbLDL-C, s-HDL-C and lower levels of l-HDL-C compared to controls.

As expected, FH subjects presented higher level of TC and LDL-C than controls, with a parallel increase in both sdLDL-C and lbLDL-C without significant differences in their percentage distribution. So, the CVD risk characteristic of the FH condition, appears to be related to the total amount of LDL-C rather than to an increase in a particular atherogenic LDL subfraction. However, when expressed as absolute figures, both lbLDL and sdLDL are significantly higher in FH group compared to the controls, suggesting that an increase in LDL-C in individuals affects by FH is paralleled by an increase in both subfractions, in agreement with literature where other authors found an association between CHD and both sdLDL-C and lbLDL-C subfractions^18^. Lipid-lowering therapy appears to be associated with lower levels of the lbLDL subfration and lower, albeit not statistically significant, levels of sdLDL in treated versus naïve FH patients^19^.

In terms of the lipid profile, no differences were detected with regard to TC, LDL-C and LDL subfractions when FH patients were stratified according to their CVD history. The lack of differences may be dependent on the fact that patients with previous CVD events should adhere to a more aggressive hypolipidemic therapy than FH-CHD- subjects. This may have prevented differences between these two groups from becoming manifest. Nevertheless, LDL mean size was lower in FH-CVD+ than in control and FH-CVD−, underling a more atherogenic lipid profile in those who experienced a CVD event^20,21^.

In our study, FH subjects and controls did not significantly differ in terms of HDL-C, while other authors reported lower levels of HDL-C in FH subjects^22–24^. Despite this, we observed differences relatively to HDL-C subfractions, supporting the paradigm that HDL quality and not only quantity is a key discriminant in dictating the beneficial effects of these lipoproteins on cardiovascular health^25^. Indeed, FH subjects presented lower levels of l-HDL-C and higher levels of s-HDL-C, which is in line with previously published data^26–28^. While l-HDL has been associated with the cardioprotective effect, s-HDL subfractions, instead, have a controversial role in CVD risk, with some reports suggesting a detrimental effect^21,24^ while others advocating a protective role against CVD^29,30^. Nevertheless in most cases, an inverse relationship between HDL size and CVD risk has been reported, whereas, some evidence suggest a possible role of s-HDL particle in dampening RCT observed in FH subjects^26^.

CEC is an in vitro surrogate assay of the first step of RCT. Some authors have detected lower CEC in FH subjects relative to controls^31,32^. Instead, in our study, FH subjects, independently of their therapy or CVD history, showed a higher CEC (both aq-CEC and ABCA1-CEC) than controls. Versmissan et al. observed an increase of CEC, in FH-CHD− subjects compared with control subjects, but not between FH-CHD+ and their healthy counterparts^33^. Asztalos et al., have described higher total CEC in CVD patients versus controls due to an increase of ABCA1-mediated CEC and pre-β1-HDL^34^. In fact, we found a clear rise in s-HDL and ABCA1-CEC in FH subjects relative to controls. However, these parameters were not correlated, which is in agreement with some studies^33,35^, instead Bellanger et al., observed an association between HDL subfractions and CEC as well as RCT in FH patients with a low HDL-C^31^. Thus, CEC in FH subjects remains a matter of contention, with conflictual results being reported in the literature. These discrepancies may be due to multiple factors which include the cell model used to assess CEC (J774, THP.1 and monocyte among others) and the pathway tested (aqueous diffusion, ABCA1- ABGC1-, or SR-BI-mediated CEC). Finally, the age and sex of the subjects whose HDL CEC was investigated, represent other variables able to influence CEC^36,37^.

It can be speculated that, aq-CEC and ABCA1-CEC are increased in FH subjects as an attempt to counterbalance hypercholesterolemia^38^. However, the existence of this compensatory mechanism remains to be proven along with its ability to compensate for the impaired lipid profile of FH subjects. Nevertheless, according to the data presented herein, this compensatory response is unlikely to suffice in order to mitigate the deleterious effect of the circulating lipid profile of FH individuals. Indeed, after normalizing for LDL-C, aq-CEC was similar for both groups, and LDL-C normalized ABCA1-CEC was even significantly lower in FH versus control group.

Of note, it has been reported that also other pathways of CEC are impaired in FH subjects, such as SR-BI or ABCG1 cholesterol efflux^31,36^, as well as other steps of RCT including cholesterol ester exchanges between HDL and apoliprotein B-containing lipoproteins as well as hepatic HDL-C uptake^31^. Moreover, a dysfunctional LDL receptor, with a consequent reduction of LDL clearance, is the most common cause of FH^2^. LDL-LDL receptor interaction and LDL clearance has been demonstrated to be a fundamental pathway of RCT^39^ indicating that independently of the increase in HDL mediated CEC RCT in FH patients remains defective due to LDL receptor dysfunction.

Despite its novelty and potential contribution to the field, this study has some limitations. First this is a retrospective study that include patients both on lipid lowering therapy as well as naïve and has a small sample size. Nevertheless, our results did not show statistical differences between these groups, which however may be due to the low number of naïve patients therefore we cannot exclude a role of hypocholesterolemic drugs in influencing the results. The same may hold true when comparing FH-CVD+ and FH-CVD−, with the use of beta-adrenergic blocking agents by the former group potentially influencing lipid metabolism. Secondly, we were unable to evaluate SR-BI and ABCG1-mediated CEC to measure total CEC. The contribution of SR-B1 is small, instead ABCG1 could contribute to about 20% of the total CEC^40^.

## Conclusions

In this study, FH subjects showed a worse metabolic profile compared to controls, characterized by a hypercholesterolemia with a shift from large to small HDL subclasses. Subjects with FH presented increased CEC compared with control subjects, which was negated after normalizing for LDL-C. LDL mean size was lower in FH subjects with a CVD history than in the controls and FH subjects without previous event. In consideration of this, monitoring the mean size of LDL and HDL subfractions could be useful to identify patients with higher CVD risk and potentially help the diagnosis of FH which still remains underdiagnosed^41^.

In light on these results, future investigations should assess the functionality of s-HDL isolated by the lipoprint system and evaluate whether the increase in CEC observed in FH patients leads to an actual increase in RTC. These findings, in turn, will shed the light on whether s-HDL and CEC may represent potential therapeutic targets for FH.

## Materials and methods

The study was conducted in accordance with the Declaration of Helsinki for studies involving humans and approved by the Ethics Committee of Ferrara (protocol code number 080392)”. Informed consent was obtained from all subjects involved in the study.

### Study design

We evaluated a cohort of adult FH subjects, stratified for presence or absence of prior CVD events, in comparison to normolipidemic subjects matched for age, sex and BMI.

FH patients and controls underwent clinical and anthropometric examination and blood testing. This study complied with the Declaration of Helsinki and was approved by the Local Ethics Committee. All subjects provided written informed consent and no personal information was available to Authors (blinding).

#### FH group

Among the subjects attending the Metabolic Unit at the University Hospital of Ferrara, 60 FH patients were identified. After a presumptive diagnosis established through Dutch Lipid Clinic Network Criteria score > 6^42^, genetic analysis of LDL-receptor, ApoB and PCSK9 genes was performed and 40 positive subjects not treated with hypocholesterolemic drugs or on stable treatment for at least 6 months (naïve, N = 7 and lipid-lowering drug-treated patients, N = 33, respectively) were recruited for the study. FH patients with previous CVD events (FH-CVD+, N = 11) were taking: lipid-lowering, beta-adrenergic blocking agents and antiplatelet drugs. Genetic mutations of FH patients are described in Supplementary Table 5S.

### Control group

80 subjects were matched by sex, age and BMI to FH patients (2:1). All subjects were in good health, none had concomitants diseases (diabetes, renal failure or cardiovascular disease) and were not taking obesity medications.

Exclusion criteria were the presence of clinically relevant secondary dyslipidemias, cancer, autoimmune or inflammatory diseases, severe psychiatric disorders, hypothyroidism, pregnancy, alcohol consumption > 10 g daily, and hormone replacement therapy.

### Biochemical analysis

Blood samples were collected after an overnight fasting. Serum or EDTA-plasma were aliquoted and stored at − 80 °C until use. Total cholesterol (Total-C), HDL-Cholesterol HDL-C), triglycerides (TG) and glucose were assayed by standard enzymatic-colorimetric methods; LDL-Cholesterol (LDL-C) was calculated according to the Friedewald formula.

### Quantification of lipoprotein subfractions

Lipoprotein subfraction analysis was performed in serum using the Lipoprint System (Quantimetrix Corporation, Redondo Beach, CA, USA) according to the manufacture’s manual. Briefly, lipoprotein subfractions were separated on the basis of size by electrophoresis on polyacrylamide gel. After the subfraction separations, gels were analyzed with Lipoware, a software that calculates the levels of cholesterol in 10 or 7 varieties for HDL or LDL kit, respectively. LDL Lipoprint kit is approved by FDA. According to the particle size, LDL particles larger than 268 Å was classified as lbLDL, while LDL particles smaller than 268 Å was referred to as sdLDL. HDL kit can quantify 10 subfractions (HDL1-HDL10) which were classified as large: 1–3 types (l-HDL); intermediate: 4–7 types (Medium HDL); small: 8–10 (s-HDL).

### Serum HDL cholesterol efflux capacity

Two pathways of cholesterol efflux were evaluated: aqueous diffusion (aq-CEC), a passive and spontaneous process and the efflux mediated by the cholesterol transporter ATP-binding cassette A1 (ABCA1) (ABCA1-CEC). Briefly, J774 mouse macrophages, cultured in DMEM containing 10% FCS, and 1% penicillin–streptomycin (Thermo Fisher Scientific, Carlsbad, CA) were labeled with [1,2–^3^H]-cholesterol in the presence of a cholesterol esterification inhibitor acyl-CoA enzyme (2 µg/ml, Sandoz 58035; Sigma-Aldrich, Milano, Italy). For ABCA1-CEC, cells were treated with 0.3 mM cAMP analogue (cpt-AMP; Sigma-Aldrich, Milan, Italy) in 0.2% BSA for 18 h to upregulate ABCA1. After washing, J774 cells were incubated with DMEM containing HDL previously isolated from serum using polyethylene glycol. CEC was expressed as a percentage of the radioactivity released to the medium after 4 h of incubation over the total radioactivity incorporated by cells. The experiments were performed in triplicate^43^. aq-CEC was detected under basal conditions, while ABCA1-CEC was the difference between values obtained with cAMP-pretreated J774 and under basal conditions. In each experiment, a reference standard containing a pool of serum was analyzed to normalize the CEC values.

### Statistical analysis

Continuous variables were expressed as mean ± standard deviation (SD) and Median (Quartile 1-Quartile 3) and analyzed for normal distribution using the Kolmogorov–Smirnov tests. Means were compared by T-test or one-way ANOVA followed by Bonferroni test or Dunnett T3 post hoc tests, whereas medians from not normally distributed variables, were compared using the nonparametric Mann Whitney or Kruskal–Wallis test. Categorical variables were expressed as the number (percentage) and analyzed with Fisher or Pearson Chi-Square Tests. Statistical analysis was performed using SPSS 22.0 software (SPSS, Chicago, IL) and a p < 0.05 was considered statistically significant.

### Ethics declarations

The study was conducted in accordance with the Declaration of Helsinki for studies involving humans and approved by the Ethics Committee of Ferrara (protocol code number 080392)”. Informed consent was obtained from all subjects involved in the study.

## Supplementary Information

Supplementary Tables.

## Data Availability

The datasets used and/or analyzed during the current study are available from the corresponding author on reasonable request.
