# Supplementary material for: Cholesterol efflux capacity is increased in subjects with familial hypercholesterolemia in a retrospective case–control study
Source: Sci Rep. 2023 May 24;13:8415. doi: 10.1038/s41598-023-35357-4 (PMC10209072; doi:10.1038/s41598-023-35357-4)
Supplement: Supplementary file 1 — Supplementary Tables. [file 41598_2023_35357_MOESM1_ESM.docx]

Cholesterol Efflux Capacity is increased in subjects with Familial Hypercholesterolemia in a retrospective case-control study

Juana Maria Sanz^1^#, Andrea D’Amuri^2^#, Domenico Sergi^3^, Sharon Angelini^3^, Valeria Fortunato^3^, Elda Favari^4^, Giovanni Vigna^5^, Giovanni Zuliani^2,3^, Edoardo Dalla Nora^2*^ and Angelina Passaro ^3,6^*

^1^ Department of Chemical and Pharmaceutical Sciences, University of Ferrara, Via Luigi Borsari, 46 - 44121 Ferrara; [juana.sanz@unife.it](mailto:juana.sanz@unife.it).

^2^ Medical Department, University Hospital of Ferrara Arcispedale Sant’Anna, Via Aldo Moro, 8 – 44124, Cona, Ferrara, Italy; [andrea.damuri@unife.it](mailto:andrea.damuri@unife.it); [giovanni.zuliani@unife.it](mailto:giovanni.zuliani@unife.it); [edoardo.dallanora@unife.it](mailto:edoardo.dallanora@unife.it).

^3^  Department of Translational Medicine, University of Ferrara, Via Luigi Borsari, 46 – 44121, Ferrara Italy; [domenico.sergi@unife.it](mailto:domenico.sergi@unife.it);

[sharon.angelini@edu.unife.it](mailto:sharon.angelini@edu.unife.it); [valeria.fortunato@unife.it](mailto:valeria.fortunato@unife.it); [giovanni.zuliani@unife.it](mailto:giovanni.zuliani@unife.it); [angelina.passaro@unife.it](mailto:angelina.passaro@unife.it)angelina.passaro@unife.it.

^4^ Department of Food and Drug, University of Parma, viale delle Scienze 27/A – 43124, Parma, Italy; [elda.favari@unipr.it](mailto:elda.favari@unipr.it)

^5^  Medicina Generale, Ospedale di Trecenta, via U. Grisetti, 265 – 45027, Trecenta (RO); [giovanni.vigna@aulss5.veneto.it](mailto:giovanni.vigna@aulss5.veneto.it).

^6^  Research and Innovation Section, University Hospital of Ferrara Arcispedale Sant’Anna, Via Aldo Moro, 8 – 44124, Cona, Ferrara, Italy;

[angelina.passaro@unife.it](mailto:angelina.passaro@unife.it).

***** Correspondence: [angelina.passaro@unife.it](mailto:angelina.passaro@unife.it); [edoardo.dallanora@unife.it](mailto:edoardo.dallanora@unife.it).

**Table 1S.** Lipid profile in naïve or lipid-lowering drug-treated FH subjects.

|  | Naïve | | Lipid-lowering drugs treated | | P value |
| --- | --- | --- | --- | --- | --- |
|  | **Mean±SD** | **Median (Q1-Q3)** | **Mean ± SD** | **Median (Q1-Q3)** |  |
| Total-C (mg/dl) | 303.3 ± 26.6 | 312.0 (289.0-324.0) | 259.5 ± 51.0 | 244.0 (222.5-287.0) | **0.035** |
| LDL-C (mg/dl) | 223.6 ± 43.3 | 231.5 (182.3-270.7) | 180.8 ± 44.2 | 173.2 (153.4-204.3) | **0.025** |
| HDL-C (mg/dl) | 58.7 ± 28.9 | 47.9 (37.1-69.1) | 52.4 ± 17.0 | 52.0 (37.0-62.6) | 0.440 |
| TG (mg/dl) | 104.7 ± 66.2 | 72.0 (66.0-152.0) | 131.2 ± 90.2 | 111.0 (73.5-140.5) | 0.326^§^ |

HDL-C, High Density Lipiprotein Cholesterol; TG, Triglycerides; SD, Standard Deviation. Means compared by T-test, whereas medians from not normally distributed variables, were compared using the nonparametric Mann Whitney test§. Naïve, N=7; Lipid-lowering drugs treated, N=33.

**Table 2S.** LDL and HDL subfraction in naïve and lipid-lowering drug-treated FH subjects.

|  | Naïve | | Lipid-lowering drugs treated | | P value |
| --- | --- | --- | --- | --- | --- |
|  | **Mean±SD** | **Median (Q1-Q3)** | **Mean±SD** | **Median (Q1-Q3)** |  |
| sdLDL (%) | 6.8 ± 6.4 | 4.3 (2.9-8.3) | 7.6 ± 9.3 | 4.0 (2.7-7.6) | 0.806§ |
| lbLDL (%) | 59.2 ± 8.0 | 60.1 (55.4-67.4) | 54.7 ± 10.7 | 55.7 (51.6-61.8) | 0.302 |
| sdLDL (mg/dl) | 13.3 ± 11.1 | 8.4 (5.0-17.3) | 13.9 ± 21.3 | 7.1 (3.8-15.0) | 0.421§ |
| lbLDL (mg/dl) | 120.7 ± 25.9 | 120.5 (103.9-143.9) | 94.1 ± 26.6 | 87.1 (75.4-112.1) | **0.021** |
| LDL size (Å) | 267.3 ± 3.9 | 268.0 (265.0-271.0) | 267.1 ± 5.6 | 268.0 (266.0-271.0) | 0.702§ |
| l-HDL (%) | 35.6 ± 14.5 | 35.9 (24.9-43.2) | 29.5 ± 10.4 | 27.2 (23.4-36.8) | 0.193 |
| m-HDL (%) | 42.3 ± 3.8 | 41.2 (40.1-45.6) | 47.0 ± 4.1 | 46.9 (43.2-50.0) | **0.009** |
| s-HDL (%) | 22.1 ± 11.1 | 21.3 (15.6-29.5) | 23.6 ± 8.8 | 23.4 (18.1-31.7) | 0.699 |
| l-HDL (mg/dl) | 21.5 ± 14.6 | 17.2 (13.6-29.4) | 16.3 ± 9.6 | 14.3 (8.9-22.1) | 0.242 |
| m-HDL (mg/dl) | 24.8 ± 11.9 | 20.5 (16.4-30.6) | 24.4 ± 7.3 | 24.1 (18.2-28.0) | 0.907 |
| s-HDL (mg/dl) | 12.4 ± 6.0 | 11.9 (10.2-18.1) | 11.8 ± 4.4 | 12.2 (9.0-14.6) | 0.730 |

FH, Familial Hypercholesterolemia; LDL, Low Density Lipoprotein; HDL, High Density Lipoprotein; sdLDL, small dense LDL; lbLDL, large buoyant LDL; l-HDL, large HDL; m-HDL, medium HDL; s-HDL, small HDL; SD, Standard Deviation. Means were compared by T-test, whereas medians from not normally distributed variables, were compared using the nonparametric Mann Whitney test§. Naïve, N=7; Lipid-lowering drugs treated, N=33.

**Table 3S.** Cholesterol Efflux Capacity in naïve and lipid-lowering drug-treated FH subjects.

|  | Naïve FH | | Lipid-lowering drug-treated FH | |  |
| --- | --- | --- | --- | --- | --- |
|  | **Mean±SD** | **Median (Q1-Q3)** | **Mean±SD** | **Median (Q1-Q3)** | **P value** |
| aq-CEC(%) | 7.8 ± 1.3 | 8.3 (6.6-8.8) | 8.5 ± 1.2 | 8.4 (7.6-9.3) | 0.260 |
| ABCA1-CEC(%) | 5.1 ± 0.7 | 5.0 (4.5-6.0) | 4.8 ± 1.3 | 5.0 (4.0-5.8) | 0.530 |
| LDL-C norm. aq-CEC | 0.034 ± 0.004 | 0.033 (0.031-0.037) | 0.050 ± 0.011 | 0.048 (0.042-0.057) | **0.001** |
| LDL-C norm. ABCA1-CEC | 0.022 ± 0.004 | 0.022 (0.019-0.024) | 0.029 ± 0.012 | 0.028 (0.022-0.033) | 0.191 |

FH, Familial Hypercholesterolemia; aq-CEC, aqueous diffusion Cholesterol Efflux Capacity; ABCA1-CEC, ATP Binding Cassette Cholesterol Efflux Capacity; LDL-C normalized aq-CEC, Low Density Lipoprotein normalized aqueous diffusion Cholesterol Efflux Capacity; LDL-C norm. (normalized) ABCA1-CEC, Low Density Lipoprotein norm. (normalized) ATP Binding Cassette Cholesterol Efflux Capacity. Means were compared by T-test. Naïve, N=7; Lipid-lowering drugs treated, N=33.

**Table 4S.** Correlation analysis between Cholesterol Efflux Capacity and lipidomic parameters from FH subjects

|  | aq-CEC (%) | | ABCA1-CEC (%) | |
| --- | --- | --- | --- | --- |
|  | **Pearson’s r** | **P value** | **Pearson’s r** | **P value** |
| Total-C (mg/dl) | 0.344 | 0.037 | -0.231 | 0.169 |
| LDL-C (mg/dl) | 0.291 | 0.080 | -0.219 | 0.194 |
| HDL-C (mg/dl) | -0.143 | 0.398 | 0.056 | 0.742 |
| TG (mg/dl) | 0.388 | 0.018 | -0.143 | 0.399 |
| sdLDL (mg/dl) | 0.200 | 0.236 | -0.022 | 0.899 |
| lbLDL (mg/dl) | 0.011 | 0.947 | -0.088 | 0.605 |
| LDL size (nm) | -0.202 | 0.230 | 0.015 | 0.930 |
| l-HDL (mg/dl) | -0.135 | 0.425 | 0.061 | 0.719 |
| m-HDL (mg/dl) | -0.078 | 0.645 | 0.008 | 0.961 |
| s-HDL (mg/dl) | -0.143 | 0.400 | 0.075 | 0.661 |

FH, Familial Hypercholesterolemia; aq-CEC, Aqueous diffusion Cholesterol Efflux Capacity; To-tal-C, total cholesterol; LDL-C, Low Density Lipoprotein Cholestero; HDL-C, High Density Lipiprotein Cholesterol; TG, Triglycerides; sdLDL, small dense LDL; lbLDL, large buoyant LDL; l-HDL, large HDL; m-HDL, medium HDL; s-HDL, small HDL; Pearson’s r, Pearson correlation coefficient. Naïve, N=7; Lipid-lowering drugs treated, N=33.

**Table 5S.** Genetic mutation of FH patients.

| N | Genetic Mutation Description | Mutation | Clinical significance |
| --- | --- | --- | --- |
| 1 | c.58 G>A, G-2R (p.G20R) | additional silent variant | Unknown effect |
| 1 | c.1567 G>A, V502M (p.V523M) | FH Bari-2 | Pathogenic |
| 1 | c.1257 C>G, Y398X (p.Y419X) | FH Caltanissetta-2 | Pathogenic |
| 1 | del G207, ins 206-208 C-L | FH Chieti+ others | Pathogenic |
| 2 | c.829 G>A, E256K (p.E277K) | FH Genova-3 | Pathogenic |
| 8 | c.682 G>A, E207K (p.E228K) | FH Modena | Pathogenic |
| 1 | c.1135 T>C, C358R (p.C379R) | FH Napoli-1, | Pathogenic |
| 2 | c.2312-3 C>A (IVS15-3 C>A) | FH Napoli-8 | Pathogenic |
| 1 | c.2416-2417 ins 1 bp (G) (V785GFsX11) | FH Napoli-9 | Pathogenic |
| 7 | c.662A>G, D200G (p.D221G) | FH Padova-1 | Pathogenic |
| 1 | c.1570 G>A, V503M (p.V524M) | FH Reggio Calabria-3 | Pathogenic |
| 1 | c.681 C>G, D206E (p.D227E) | FH Reggio Emilia-2 | Pathogenic |
| 1 | c.1775 G>A, G571E (p.G592E) | FH Sicily | Pathogenic |
| 1 | c.1775 G>A, G571E (p.G592E)  + c.1706 G>A (IVS11-10 G>A) | FH Sicily + other | Pathogenic |
| 1 | c.10672 C>T, R3531C (p.R3558C) | ApoB mutation | Pathogenic |
| 1 | c.746 T>C, I228T (p.I249T) | New sequence variant | Unknown effect |
| 2 | c.1306 del G, E414FsX15 | New sequence variant | Unknown effect |
| 1 | c.1977 C>A, T638T (p.T659T) | New sequence variant | Unknown effect |
| 1 | c.2364 G>T, R767S (p.R788S)) | New sequence variant | Unknown effect |
| 1 | multiple intronic variants | New sequence variants | Unknown effect |
| 1 | c.1428_1429 ins 1bp (C) P455FsX59 | New sequence variant | Unknown effect |
| 1 | c.1747 C>G, H562D (p.H583D) | New sequence variant | Unknown effect |
| 1 | c.2390-2 A>G (IVS16-2 A>G) | New sequence variants | Unknown effect |

FH, Familial Hypercholesterolemia
